# Multiplex real time PCR panels to identify fourteen colonization factors of enterotoxigenic *Escherichia coli* (ETEC)

**DOI:** 10.1371/journal.pone.0176882

**Published:** 2017-05-05

**Authors:** Jie Liu, Sasikorn Silapong, Pimmada Jeanwattanalert, Paphavee Lertsehtakarn, Ladaporn Bodhidatta, Brett Swierczewski, Carl Mason, Annette L. McVeigh, Stephen J. Savarino, Rosemary Nshama, Esto Mduma, Athanasia Maro, Jixian Zhang, Jean Gratz, Eric R. Houpt

**Affiliations:** 1 Division of Infectious Diseases and International Health, University of Virginia, Charlottesville, Virginia, United States of America; 2 Armed Forces Research Institute of Medical Sciences (AFRIMS), Bangkok, Thailand; 3 Infectious Disease Clinical Research Program (IDCRP), Enteric Disease Department, Infectious Disease Directorate, Naval Medical Research Center, Silver Spring, Maryland, United States of America; 4 Haydom Global Health Centre, Haydom, Tanzania; 5 Kilimanjaro Clinical Research Institute, Moshi, Tanzania; Seconda Universita degli Studi di Napoli, ITALY

## Abstract

Enterotoxigenic *Escherichia coli* (ETEC) is a leading cause of childhood diarrhea in low income countries and in travelers to those areas. Inactivated enterotoxins and colonization factors (CFs) are leading vaccine candidates, therefore it is important to determine the prevailing CF types in different geographic locations and populations. Here we developed real time PCR (qPCR) assays for 14 colonization factors, including the common vaccine targets. These assays, along with three enterotoxin targets (STh, STp, and LT) were formulated into three 5-plex qPCR panels, and validated on 120 ETEC isolates and 74 *E*. *coli* colony pools. The overall sensitivity and specificity was 99% (199/202) and 99% (2497/2514), respectively, compared to the CF results obtained with conventional PCR. Amplicon sequencing of discrepant samples revealed that the qPCR was 100% accurate. qPCR panels were also performed on nucleic acid extracted from stool and compared to the results of the ETEC isolates or E. coli colony pools cultured from them. 95% (105/110) of the CF detections in the cultures were confirmed in the stool. Additionally, direct testing of stool yielded 30 more CF detections. Among 74 randomly selected *E*. *coli* colony pools with paired stool, at least one CF was detected in 63% (32/51) of the colony pools while at least one CF was detected in 78% (47/60) of the stool samples (P = NS). We conclude that these ETEC CF assays can be used on both cultures and stool samples to facilitate better understanding of CF distribution for ETEC epidemiology and vaccine development.

## Introduction

Enterotoxigenic Escherichia coli is a leading cause of diarrhea in children of developing countries [1, 2] and in travelers to those areas [3]. ETEC are defined by their ability to produce a heat-labile toxin (LT) and/or a heat-stable toxin (ST). ST from human ETEC strains consists of two variants STh and STp, also known as ST-Ia and ST-Ib, respectively. To cause diarrhea, ETEC toxins phosphorylate cystic fibrosis transmembrane conductance regulator, which activates its chloride channel and leads to efflux of chloride into the intestinal lumen. First, however, strains must adhere to small bowel enterocytes, a process mediated by the presence of one or more surface fimbriae termed colonization factors (CF). More than 25 ETEC CFs are recognized, each made up of hundreds of copies of one or more subunit proteins. The toxin and colonization factor genes are acquired via common or distinct plasmids. Multilocus sequence typing and whole genome sequencing has demonstrated that ETEC are genetically diverse across various *E*. *coli* phylogroups [4–6], however a discrete number of lineages exist that are marked by specific CF and enterotoxin profiles [6]. Thus certain CF-toxin combinations are common, for example CFA/I with STh, CS1 and CS3 with LT and STh, CS17 and LT, and CS6 with STp [6, 7].

Defining the epidemiology of CFs worldwide has important implications for modern ETEC vaccine candidates. The general vaccine strategy involves the inclusion of multiple CFs with an LT component to provide broad coverage (ST has generally been toxic and poorly immunogenic). Leading candidates include inactivated strains overexpressing CFA/I, CS3, CS5, and CS6 mixed with an LT B-subunit related toxoid and adjuvant (ETVAX) and a 3 component live attenuated ETEC that expresses CFA/I, CS1, CS2, CS3, CS5, CS6 and the B subunit of LT (ACE527) [8, 9]. A recent review estimated that such vaccines should cover 60–80% of ETEC circulating globally [10]. However, these estimates vary by region, time, travelers versus children, and have been confounded by variability in CF detection methodologies. Indeed, approximately a third of ETEC isolates have no CF identified [10].

Historically, phenotypic methods such as immunoassays have been used for CF typing, using polyclonal or monoclonal antibodies against various epitopes [7]. These methods are not user friendly, particularly for field settings, and commercial sources of reagents are limited. Therefore molecular methods have been developed, including DNA hybridization and singleplex and multiplex PCR assays followed by gel electrophoresis detection [11, 12]. Real time PCR (qPCR) methods have excellent performance characteristics and minimize contamination but have not been described, except for an assay for CS6 identification [13]. A test that could be performed directly on stool would be ideal, since culturing of *E*. *coli* is cumbersome and may easily lead to loss of CFs [14]. Therefore, here we undertook the development and validation of three panels of TaqMan probe based real time PCR assays to detect fourteen major colonization factors along with the enterotoxins, and evaluated performance on both cultures and stool.

## Materials and methods

### Specimens

120 ETEC isolates tested in this work included 108 clinical isolates chosen to obtain a distribution of CF expression and 12 laboratory strains: H10407 (CFA/I), E24377A (CS1/PCF071, CS3), B2C (CS2, CS3, CS21), BANG10-SP (CS4, CS6), ETEC 8/11 (CS5, CS6), D02-2 (CS7), E25281C (CS6, CS8), 350C1A (CS12), WS3294A (CS14), WS0115A (CS17/CS19), ARG-2 (CS18), WS7179A-2 (CS20). All were previously characterized by multiplex PCR followed by gel electrophoresis using assays of Nada et al. and/or Rodas et al. [11, 12]. We also obtained stool specimens and *E*. *coli* cultures from a study on The Etiology, Risk Factors and Interactions of Enteric Infections and Malnutrition and the Consequences for Child Health and Development (MAL-ED). In this study, *E*. *coli* cultures were stored as either a pool of 5 colonies or individual isolates. ETEC was confirmed by PCR of ST and LT as described [15]. For this work we obtained 50 paired ETEC isolate and stool samples from the Nepal site to cover a range of CF types and randomly selected 74 paired E. coli colony pools and stool from the Tanzania site, including 14 ETEC negative samples. These 124 MALED stools included 96 non-diarrheal stools and 28 diarrheal stools, including 8 mild, 15 moderate, and 5 severe episodes [2]. The University of Virginia and local Institutional Review Boards approved this work. Written informed consent was obtained from the parent or guardian of every child.

### Nucleic acid extraction

One milliliter of overnight cultures (14–16 hr) of each isolate was resuspended in TE buffer (10 mM Tris-HCl, pH 8.0) and boiled for 20 min. The supernatant was collected after a 5 minute spin at 12,000 x g and used directly for PCR. Nucleic acid was extracted from 200 mg of stool samples using the QIAamp Fast Stool DNA mini kit with pretreatments and spiked with external controls as described previously [16].

### PCR design

Real time PCR primers and probes targeting the colonization factors were designed based on the subunit genes and amplicons described previously [11]. BLAST was performed to assure 100% in silico specificity. Primers and probes were procured from Integrated DNA Technologies (Coralville, IA), and LGC BioSearch Technologies (Petaluma, CA). Three 5-plex real time PCR panels were formulated as shown in Table 1. STh, STp, and LT assays were adapted from our previous work [17].

**Table 1:** Primer and probe sequences of the real time PCR panels.

Panel	Target	Gene	Sequence (F: forward, R: reverse, P: probe)	Concentration (nM)	Amplicon length(bp)
I	CFA/I	*cfaB*	F: AGCTTATTCTCCCGCATCAAA R: GAACATCTGTAAGCTGTGGTGT P: FAM-ACTCAAGTACATACAAACGATGCA	200 200 100	122
	CS4	*csaB*	F: CTATTCACCTGCGGCAAGTC R: GGGGAGTTGTTTTGTAGAATCCA P: Texas Red-TCGCAACTAAAGTTCATACAAATGT	300 300 150	142
	CS6	*cssB*	F: GGAGTGGTAAATGCAGGAAACT R: GAACAGCGGAATCAATATCTGGA P: HEX-CTCTGGATGTAAATGTAAATATTGAG	300 300 150	94
	CS14	*csuA1*	F: TCATGGGCAGGGAAGACATT R: TACTATTCGAAACACCTGCCG P: Quasar705-AGTTGGCGATCTGGGTTTTG	200 200 100	97
	CS18	*fotA*	F: GGTACCTTAAATGGCCAGCC R: TAACAGTACCAGCTTTAACCTGAC P: Quasar670-CGCATTGCCCAAAACACCT	200 200 100	67
II	CS1/PCFO71	*csoA*	F: ACTTTGCTTCGAGTGGTGTT R: CCCTGATATTGACCAGCTGTTA P: Quasar670-CAGAAACTTTCAATCCATGCAGAT	300 300 150	109
	CS2	*cotA*	F: TCTGCTCGTATCAATACCCAAGTT R: GTGCCAGCGAATGAAACCTCTA P: FAM-TCTGATCCAAGCAAGACTATTCC	200 200 100	140
	CS8	*cofA*	F: ACTGGGAGTATGTGGCAGTTG R: TATTGTAGTATTATCAGTAGCAGCCA P: Quasar705-CATGTTTACTGCGCCTGCA	400 400 200	101
	CS17/19	*csbA-csdA*	F: AGGSAGTTGTAGTGAAGCTGT R: GTCACTTTCATCGGAATTTGCGA P: HEX-CAGTTCTGTCCAATATTATGAAGCCA	300 300 150	82
	CS21	*lngA*	F: GGACCCATTAAGCCTTACTGC R: GTTATTACGCACTTCGTCTGGT P: Texas Red-TGCAGCACAGTTAGTTCAGC	200 200 100	109
III	CS3	*cstA*	F: GGTCTTTCACTGTCAGCTATGA R: CCAAGTTGCATCCAGAGCTG P: FAM-TGGCATTAAATGTGCTTTCTCCT	200 200 100	111
	CS5	*csfA*	F: GCGTGACACGTCAGCTAATATAAAC R: AAAGTGATTGCGACTTCCCC P: HEX-ACCGCAGTAGAAGCAGCTAA	300 300 150	138
	CS7	*csvA*	F: TGCTCCCGTTACTAAAAATACGT R: CGAACGGGCTGTGATACCTT P: Texas Red-CCAATCCGTTCACAAAAGCC	200 200 100	118
	CS12	*cswA*	F: TTACGTCTCTGATCATGGCTGTTA R: TTGTTATTCGCTTGGCCGTT P: Quasar705-ATGAATAGCTCAGCCTTCGC	300 300 150	82
	Enterotoxin	*STh*,	Fh: GCTAAACCAGYAGRGTCTTCAAAA Rh: CCCGGTACARGCAGGATTACAACA Ph: Quasar670-TGGTCCTGAAAGCATGAA	200 200 100	147
		*STp*,	Fp: TGAATCACTTGACTCTTCAAAA Rp: GGCAGGATTACAACAAAGTT Pp: Quasar670-TGAACAACACATTTTACTGCT	200 200 100	136
		*LT*	Fl: TTCCCACCGGATCACCAA Rl: CAACCTTGTGGTGCATGATGA Pl: Quasar670-CTTGGAGAGAAGAACCCT	200 200 100	62

### PCR conditions

Each 20 μl reaction included 10 μl of IQ Multiplex Powermix (BioRad, Hercules, CA), 1 μl of cell lysate or stool extract, and primers and probes at the concentrations indicated in Table 1. Cycling conditions included 3 min of initial denaturation at 95°C and 40 cycles of 15 sec at 95°C and 1 min at 60°C. Real time PCRs were performed on CFX real time PCR system (BioRad), and the data were analyzed with BioRad CFX Manager 3.1. A quantitative cycle (Cq, inversely related to target quantity) cutoff of 35 was set for calling positivity as per our previous work. One pooled positive control and one No-Template-Control was included on each plate. All control results were valid.

### Assay validation

Linearity, precision, lower limit of detection and specificity were tested for all three panels. Linearity, precision, lower limit of detection were measured using the strains listed in the S1 Table. Specificity was tested against a panel of enteropathogens, as listed in the S2 Table.

### Assay performance

The real time PCR assays were performed on the ETEC isolates and E. coli colony pools along with the corresponding stool samples. The conventional multiplex PCR gel electrophoresis based assay [11] was also performed on the E. coli colony pools. The CF results were compared between real time PCR and conventional PCR on isolates, and between isolates or E. coli colony pools and the corresponding stool. Discrepant samples were amplified with the relevant conventional PCR assays and subjected to amplicon sequencing.

### Statistics

Correlation was tested by regression analysis using the ANOVA test. Cq values were compared with the Mann-Whitney U test between isolate positive/stool positive and isolate negative/stool positive samples. Two-tailed P values were calculated, and values of < 0.05 were considered statistically significant. All analyses were performed using SPSS version 24.

## Results

### Assay performance on the ETEC isolates

The 12 reference ETEC strains included all the CF types interrogated plus CS20. Analytical performance of the 3 real time PCR panels is shown in S1 Table. The assays correctly identified all CFs with no cross-reactivity to each other or to a list of pathogens found in fecal samples (S2 Table). The lower limit of detection was between 5×10^3^ and 5×10^4^ CFU per gram of stool depending on the target.

**Fig 1:**
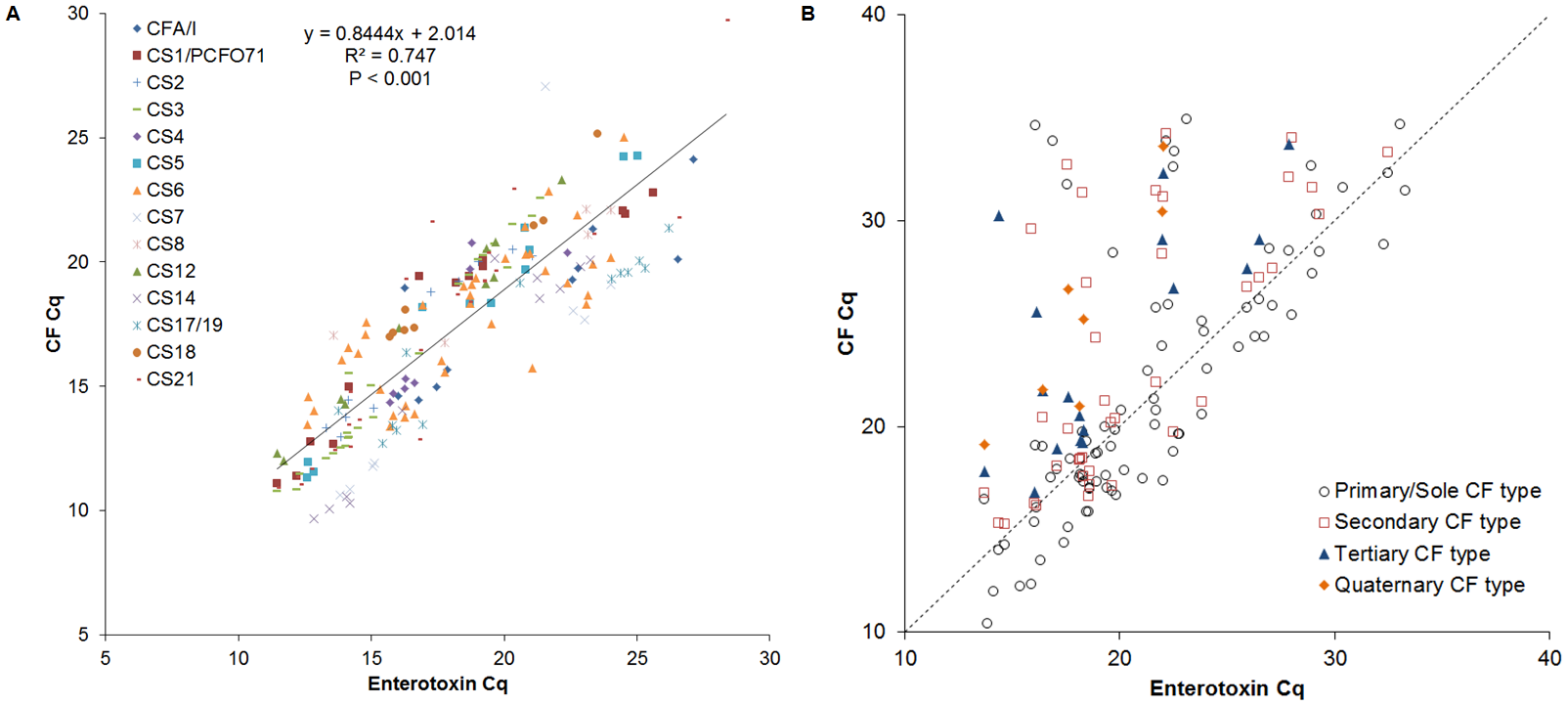
Correlation of Cqs between enterotoxin and CFs on cultures (A) and stool (B). (A) On culture (N = 194), the overall correlation R^2^ = 0.78 (*P* < 0.001). Each symbol represents one CF type. (B) On stool, the Cq correlation between enterotoxin and primary/sole CF (the most abundant CF type measured by Cq values), secondary CF (the second most abundant, if present), tertiary CF (the third most abundant, if present), and quaternary CF (the fourth abundant, if present) CF were all statistically significant (P < 0.05, with R^2^ = 0.66 (N = 81), 0.45 (N = 39), 0.46 (N = 17), and 0.82 (N = 7), respectively. The dotted line shows the diagonal line.

Real time PCR results were further compared with conventional PCR results on 108 clinical isolates and 74 E. coli colony pools. Using the conventional PCR results as the gold-standard, the sensitivity and specificity were each 99% (Table 2). The three conventional positive but real time PCR negative specimens were confirmed to be negative with the relevant singleplex conventional assays, presumably due to the loss of plasmid in the subculture. The 17 real time PCR positive but conventional (multiplex) PCR negative results were all confirmed to be positive with singleplex PCR followed by amplicon sequencing. 58% of the isolates/pools possessed one colonization factor while 42% had more than one. S1 Fig shows the distribution of various CF types. Since we selected samples on the basis of CF expression for assay validation purposes, these distributions are not epidemiologically representative. However, we can note some expected findings, such as CS3 was commonly found with CS1 or with CS2 (i.e., the CFA/II family), and CS6 was commonly found either alone or with CS4 or CS5 (i.e., the CFA/IV family). The Cq of CF had moderate correlation with Cq of the corresponding enterotoxin genes, with an overall Pearson coefficient of 0.7470 (Fig 1A).

**Table 2:** Comparison of real time PCR results with conventional PCR results on 120 ETEC isolates and 74 *E*. *coli* pools.

	Conventional PCR positive		Conventional PCR negative		qPCR	
	qPCR +	qPCR -	qPCR +	qPCR -	Sensitivity, %	Specificity, %
CFA/I	11	0	0	183	100	100
CS1/PCF071	17	0	4	173	100	98
CS2	11	0	0	183	100	100
CS3	27	0	1	166	100	99
CS4	10	0	0	184	100	100
CS5	12	0	0	182	100	100
CS6	38	0	5	151	100	97
CS7	9	0	0	185	100	100
CS8	5	0	0	189	100	100
CS12	11	0	0	183	100	100
CS14	12	0	0	182	100	100
CS17/19	14	1	0	179	94	100
CS18	4	0	0	190	100	100
CS21	18	2	7	167	90	96
Total	199	3	17	2497	99	99

### CF identification directly on stool samples

To test the feasibility of performing these real time PCR assays directly on stool samples, we compared the stool PCR results with those of the corresponding isolates or *E*. *coli* colony pools. 95% (105/110) of the CF detections in the cultures, either isolates or *E*. *coli* pools, were confirmed by the results from stool. Additionally, direct testing of stool samples yielded 30 more CF detections. Cqs were not significantly different between the culture positive/stool positive and culture negative/stool positive samples (21.2 ± 5.3 vs. 22.8 ± 4.1, P = 0.104). To adjudicate discrepancies, the relevant conventional PCR assays were performed in singleplex followed by sequencing, and all the excess stool CF detections were confirmed (data not shown). As with the isolates, the enterotoxin quantity in stool was statistically correlated with the CF types (Fig 1B, *P* < 0.05).

**Fig 2:**
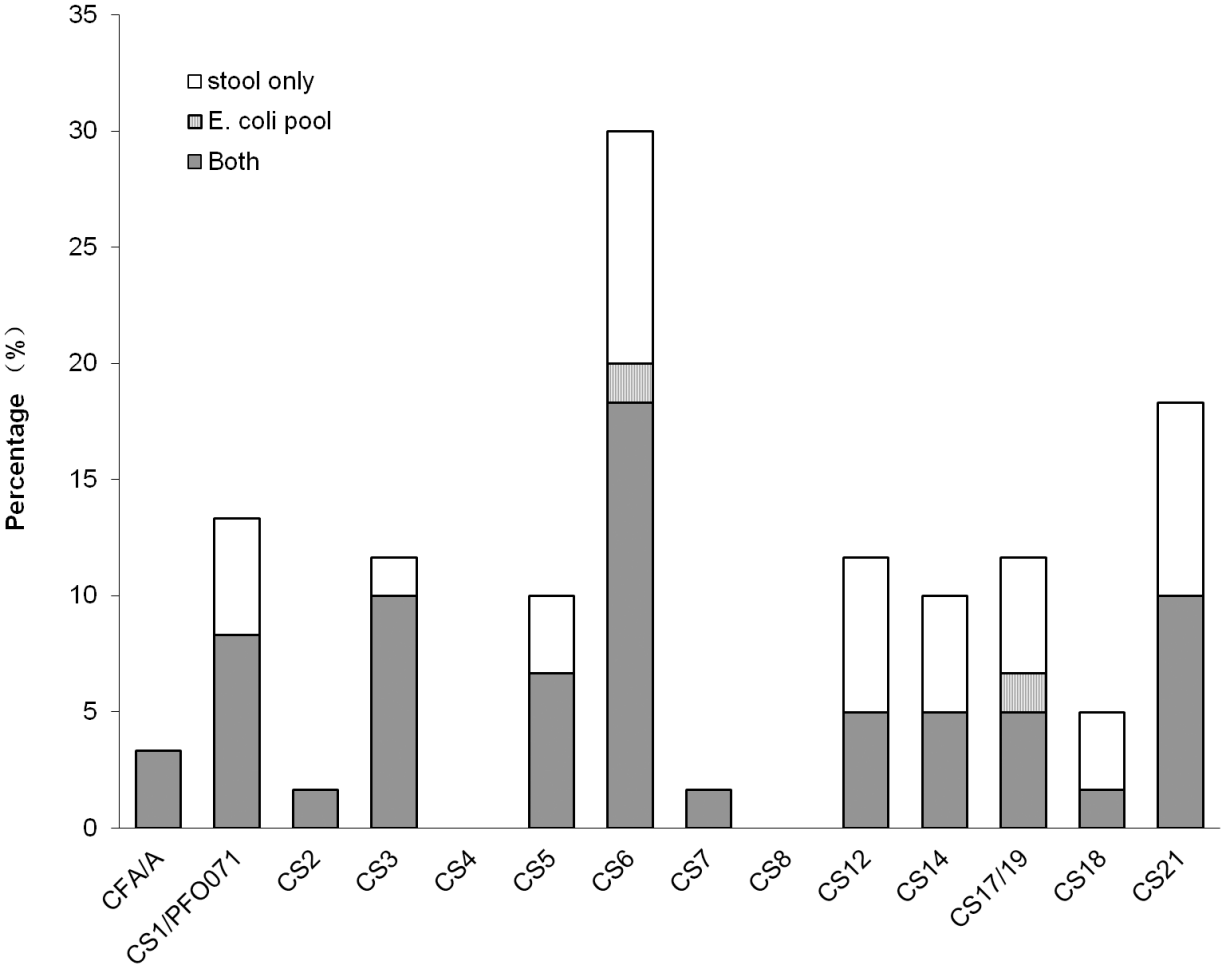
Comparison of CF detection on randomly selected stool samples and the corresponding *E*. *coli* pools.

Among the 74 randomly selected stool and paired *E*. *coli* colony pools from the Tanzania site of MAL-ED, we were able to describe the CF profile and also the CF relationship to the enterotoxins. At least one CF was detected in 63% (32/51) of these colony pools whereas at least one CF was detected in 78% (47/60) of the stool samples (Fig 2, P = NS). The additional detection seen in stool samples was spread across several CF types. With regard to toxin profile, 100% (4/4), 88% (15/17), 85% (11/13), 65% (11/17), 63% (5/8), and 100% (1/1) had CFs detected for STh+STp+LT+, STh+LT+, STp+LT+, LT only, STh only, and STp only, respectively. Though not common, 13% (2/16) of the ST-LT- stool samples had CFs detected, both with Cq > 33.

## Discussion

In this work we developed real time PCR assays for ETEC CFs that demonstrated excellent analytical and clinical performance on cultures, either individual isolates or pools of colonies. Moreover, the assay detected CFs well directly on stool, with additional detection versus that of the cultures. We believe these stool detections are true positives, given the 99% specificity of the assays on pure cultures, and likely reflects the instability of plasmids upon culture and subculture [18, 19]. Use of these assays on stool could thus serve to close the “CF-negative” ETEC gap, which is about 34% according to a large survey [6].

Another advantage of using stool is that obtaining isolates requires culture followed by picking a small and arbitrary number of colonies, whereas the stool audits the entire specimen. A disadvantage to using stool is that one cannot know for certain if the CF and enterotoxin detections are from the same ETEC or different *E*. *coli*. However the detection of CF and enterotoxin were correlated, consistent with them both deriving from the same ETEC (Fig 1). Another disadvantage to testing stool directly is that susceptibility testing cannot be readily performed. On balance we feel the advantages and ease of direct stool testing outweigh the disadvantages for large scale studies, while culture can be performed on a subset of stools for confirmatory typing and susceptibility purposes, if needed.

The primary purpose of this work was to develop a tool that could be used for ETEC surveillance and vaccine studies. ETEC, particularly those strains that produce the STh enterotoxin, are one of the world’s top 4 or 5 major causes of childhood diarrhea [1, 2, 20] and have been the leading pathogen in large traveler’s diarrhea studies [3]. ETEC vaccines are under development based on major colonization factors including CFA, CS1, CS2, CS3, CS5, and CS6 and heat labile toxin [21, 22]. Recent reviews have estimated that such vaccines should cover 60–80% of circulating ETEC globally, but these are based on results of direct isolates [10].

Our future goal is thus to use these assays to document the pattern of CFs and enterotoxins, with a guide towards vaccine coverage. It has been reported, for example, that CFA/I is more common in endemic versus travel populations [10]. It is encouraging in this regard that the pattern of CFs seen thus far, on these validation samples, followed expected norms, for example CFA/I was common ± CS21 (aka Longus), CS3 was commonly found with CS1, or with CS2 (i.e., the CFA/II family), and CS6 was commonly found either alone or with CS4 or CS5 (i.e., the CFA/IV family). Likewise, the better detection of CFs in ST±LT strains versus LT only strains that we observed (83% versus 65%) has been noted previously [6, 7]. It is also possible that clinical presentations differ among ETEC CF-containing strains in conjunction with their enterotoxin profiles [23], although this needs to be convincingly demonstrated in future work.

This study has some limitations. First, the sample size was relatively small to test the performance on the rarer CFs. Secondly, any assay design will need updating with the emerging of new CF types and variants in the targeted gene regions.

In summary, we present assays for ETEC CFs that can help illuminate the global epidemiology of this pathogen of great public health importance.

## Supporting information

S1 Fig CF type distribution in ETEC clinical isolates (N = 194) tested with real time PCR panels. (DOCX)

S1 Table Analytical performance. Linearity was tested with a 10-fold serial dilution of the corresponding material. For limit of detection and precision, stool samples from healthy donors were spiked with cultured isolates, then extracted and assayed. Intra-assay precision was tested with 10 repeats within one run and inter-assay precision was tested with 10 identically spiked samples that were extracted and assayed over 5 days. Limit of detection was defined as the lowest concentration at which the target could be detected in all 10 spiked samples.
(DOCX)

S2 Table Enteropathogens used for specificity testing. (DOCX)
