# Supplementary figures and images for: Multiplex real time PCR panels to identify fourteen colonization factors of enterotoxigenic Escherichia coli (ETEC)
Source: PLoS One. 2017 May 5;12(5):e0176882. doi: 10.1371/journal.pone.0176882 (PMC5419558; doi:10.1371/journal.pone.0176882)

S1 Fig. CF type distribution in ETEC clinical isolates (N=194) tested with real time PCR panels.
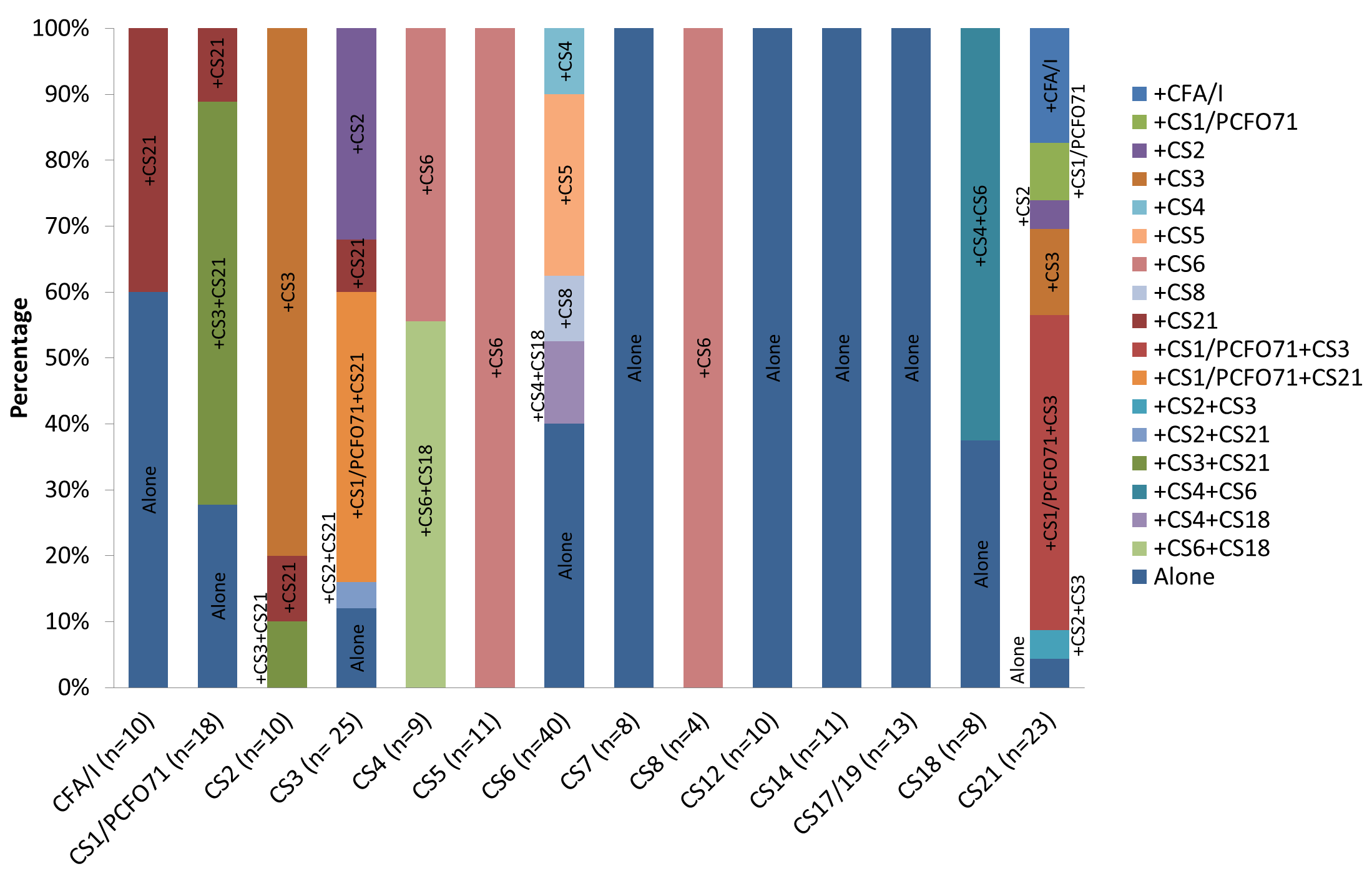

Supplement: S1 Fig — (DOCX) [file pone.0176882.s001.docx]
