# Supplementary material for: Multiplex real time PCR panels to identify fourteen colonization factors of enterotoxigenic Escherichia coli (ETEC)
Source: PLoS One. 2017 May 5;12(5):e0176882. doi: 10.1371/journal.pone.0176882 (PMC5419558; doi:10.1371/journal.pone.0176882)
Supplement: S1 Table — Linearity was tested with a 10-fold serial dilution of the corresponding material. For limit of detection and precision, stool samples from healthy donors were spiked with cultured isolates, then extracted and assayed. Intra-assay precision was tested with 10 repeats within one run and inter-assay precision was tested with 10 identically spiked samples that were extracted and assayed over 5 days. Limit of detection was defined as the lowest concentration at which the target could be detected in all 10 spiked samples. (DOCX) [file pone.0176882.s002.docx]

S1 Table. Analytical performance. Linearity was tested with a 10-fold serial dilution of the corresponding material. For limit of detection and precision, stool samples from healthy donors were spiked with cultured isolates, then extracted and assayed. Intra-assay precision was tested with 10 repeats within one run and inter-assay precision was tested with 10 identically spiked samples that were extracted and assayed over 5 days. Limit of detection was defined as the lowest concentration at which the target could be detected in all 10 spiked samples.

| Panel | Target | strain | PCR efficiency (%) | linearity | Precision (%) | | Limit of detection,  CFU/g stool (CV) |
| --- | --- | --- | --- | --- | --- | --- | --- |
|  |  |  |  |  | Intra-assay | Inter-assay |  |
| I | CFA/I | H10407 | 98.0% | 0.991 | 2.0 | 4.2 | 5×10^3^ (5.7%) |
|  | CS4 | BANG10-SP | 95.4% | 0.989 | 2.9 | 4.1 | 5×10^3^ (7.7%) |
|  | CS6 | BANG10-SP | 91.2% | 0.995 | 2.3 | 4.1 | 5×10^3^ (4.9%) |
|  | CS14 | WS3294A | 97.0% | 0.986 | 2.3 | 2.9 | 5×10^3^ (6.8%) |
|  | CS18 | ARG-2 | 91.4% | 0.979 | 2.1 | 5.1 | 5×10^4^ (5.7%) |
| II | CS1/PCFO71 | E24377A | 93.8% | 0.974 | 1.8 | 3.5 | 10^4^ (4.5%) |
|  | CS2 | B2C | 97.2% | 0.968 | 2.0 | 4.1 | 5×10^4^ (5.1%) |
|  | CS8 | E25281C | 87.4% | 0.984 | 2.6 | 3.9 | 10^4^ (6.4%) |
|  | CS17/19 | WS0115A | 96.1% | 0.975 | 1.6 | 4.8 | 5×10^3^ (8.5%) |
|  | CS21 | B2C | 92.6% | 0.987 | 1.8 | 6.5 | 10^4^ (6.8%) |
| III | CS3 | B2C | 96.6% | 0.997 | 1.5 | 4.4 | 10^4^ (5.3%) |
|  | CS5 | ETEC 8/11 | 90.6% | 0.993 | 2.1 | 5.8 | 10^4^ (7.9%) |
|  | CS7 | DO2-2 | 96.7% | 0.985 | 1.8 | 4.6 | 5×10^3^ (9.6%) |
|  | CS12 | 350C1A | 91.4% | 0.987 | 2.0 | 3.4 | 5×10^4^ (4.3%) |
|  | enterotoxin |  | 85.6% | 0.966 | 2.0 | 4.6 | 10^4^ (5.7%) |
