# Supplementary material for: Multiplex real time PCR panels to identify fourteen colonization factors of enterotoxigenic Escherichia coli (ETEC)
Source: PLoS One. 2017 May 5;12(5):e0176882. doi: 10.1371/journal.pone.0176882 (PMC5419558; doi:10.1371/journal.pone.0176882)
Supplement: S2 Table — (DOCX) [file pone.0176882.s003.docx]

S2 Table. Enteropathogens used for specificity testing.

|  | **Species** |
| --- | --- |
| Bacteria | EAEC  EPEC  STEC  EIEC  *Aeromonas hydrophila*  *Bacteroides fragilis*  *Clostridium difficile*  *Campylobacter jejuni*  *Campylobacter coli*  *Campylobacter hyointestinalis*  *Campylobacter upsalensis*  *H. pylori*  *Salmonella enterica*  *Shigella boydii*  *Shigella dysenteriae*  *Shigella flexneri*  *Shigella sonnei*  *Vibrio cholerae*  *Vibrio parahaemolyticus*  *Yersinia enterocolitica* |
| DNA virus | Adenovirus 5  Adenovirus 40  Adenovirus 41  Cytomegalovirus |
| Parasite | *Cryptosporidium hominis*  *Cryptosporidium parvum*  *E. histolytica*  *E. dispar*  *E. intestinalis*  *Giardia lamblia*  *Schistosoma mansoni* |
